# Hypertensive Emergency in the Context of SARS‐CoV‐2 Infection: Clinical Course and Management

**DOI:** 10.1155/cric/9408438

**Published:** 2026-05-06

**Authors:** Gudisa Bereda

**Affiliations:** ^1^ Department of Pharmacy, Marie Stopes International Ethiopia (MSIE), Ambo, Ethiopia

**Keywords:** antihypertensive therapy, blood pressure, COVID-19, hypertensive emergency, reflex tachycardia, SARS-CoV-2 infection

## Abstract

Coronavirus Disease2019 (COVID‐19) can increase sympathetic nervous system activity by inducing systemic inflammation and a cytokine storm. This can result in elevated blood pressure, increased heart rate, and vasoconstriction. A 67‐year‐old Black male was admitted to the emergency department with a chief complaint of shortness of breath for 2 days. On examination, he exhibited edema and symmetric palpable pulses in his lower limbs. His electrocardiogram revealed left axis deviation, sinus tachycardia, and ST‐segment elevation. Reverse transcription‐polymerase chain reaction confirmed COVID‐19. Hydralazine (5 mg) was administered intravenously immediately. The patient was re‐evaluated after 20 min during his stay in the emergency department. After two 5 mg doses of hydralazine, he developed reflex tachycardia. To control heart rate, IV hydralazine was replaced with oral atenolol (50 mg once daily), a *β*₁‐blocker that reduces cardiac output. Subcutaneous enoxaparin 80 mg daily was given to prevent thrombotic complications in hospitalized COVID‐19 patients. Acute hypertension and COVID‐19‐induced hypertensive emergencies in patients with preexisting hypertension are typically associated with a worse prognosis.

**Key Learning Points**

➢ COVID‐19 can trigger hypertensive emergencies in patients with preexisting hypertension through sympathetic overactivity, renin‐angiotensin‐aldosterone system (RAAS) dysregulation, hypoxia, and systemic inflammation.
➢ SARS‐CoV‐2 binds to ACE2 receptors, reducing the protective effects of ACE2 on blood pressure regulation and exacerbating hypertension.
➢ COVID‐19‐induced endothelial injury impairs vasodilation, increases vascular resistance, and contributes to acute blood pressure elevation.
➢ Rapid blood pressure control is critical; intravenous hydralazine effectively reduces acute BP but may cause reflex tachycardia.
➢ Combination therapy with atenolol and nifedipine safely controls both heart rate and blood pressure, preventing further cardiovascular stress.
➢ Continuous cardiac monitoring and supportive care (oxygen, anticoagulation, and antimicrobials) are essential for safe management in COVID‐19‐associated hypertensive emergencies.
➢ Long‐term follow‐up and integrated therapy planning ensure maintenance of target BP and prevention of recurrent hypertensive crises.
➢ Integration of COVID‐19 therapy considerations (e.g., drug–drug interactions, and inflammation management) is important for optimal cardiovascular outcomes.

## 1. Introduction

In 2024, approximately 1.4 billion individuals aged 30–79 years were affected by hypertension globally, but fewer than 20% achieved effective blood pressure control [1]. Hypertensive emergencies are clinical conditions characterized by acute or persistent injury to target organs and markedly elevated blood pressure, usually ≥ 180/120 mmHg [2]. The Coronavirus Disease2019 (COVID‐19) pandemic is caused by Severe Acute Respiratory Syndrome Coronavirus 2 (SARS‐CoV‐2) [3, 4]. Hypertension is a common chronic condition among patients hospitalized with COVID‐19 [5]. Acute elevations in blood pressure in COVID‐19 patients may be associated with in‐hospital mortality, intensive care unit admission, heart failure, acute end‐organ damage, and worse outcomes [6]. Physiological responses to acute respiratory distress syndrome (ARDS), including sympathetic nervous system (SNS) hyperactivity, RAAS dysregulation, and cardiovascular strain, can lead to marked increases in blood pressure [7]. However, ARDS itself does not directly cause a hypertensive emergency.

Inflammation, hypoxia, RAAS dysregulation, and iatrogenic factors may significantly worsen preexisting hypertension in patients with COVID‐19, potentially resulting in a hypertensive emergency [8]. Management of hypertensive emergency involves monitoring for end‐organ damage and rapidly controlling blood pressure with intravenous antihypertensive agents, such as labetalol or nicardipine. In addition, RAAS inhibitors, calcium channel blockers, beta‐blockers, and diuretics may be useful in managing elevated blood pressure in COVID‐19 patients. [9] This case report describes a patient with preexisting hypertension and SARS‐CoV‐2 infection who developed a hypertensive emergency.

## 2. What This Study Contributes

This study emphasizes the necessity of integrated care pathways for the management of hypertensive emergencies, with a focus on minimizing organ damage and improving patient outcomes. It also highlights the potential mechanisms underlying hypertensive emergencies in the context of COVID‐19. The findings underscore the importance of early blood pressure monitoring in patients with COVID‐19, particularly those with preexisting hypertension or other cardiovascular risk factors. Considering COVID‐19 as a contributing factor provides a more comprehensive understanding of the interaction between viral infections and chronic diseases. These findings may facilitate the early recognition and clinical management of similar cases in clinical practice.

## 3. Case Presentation

A 67‐year‐old Black male was admitted to the emergency department with a chief complaint of shortness of breath for 2 days. He reported associated symptoms of dizziness, nausea, blurred vision, and generalized weakness. Physical examination revealed edema in the lower limbs with symmetrically palpable pulses. His past medical history was significant for Stage I hypertension for the previous 5 years. He had been taking enalapril and hydrochlorothiazide for blood pressure control. He had no history of smoking, alcohol consumption, or tea intake. His blood pressure had previously been well controlled with antihypertensive medications and dietary modifications. He reported no family history of significant medical conditions. During the 2‐weeks preceding admission, the patient had no travel history. He had no previous confirmed diagnosis of COVID‐19. However, he lived with his wife, who had tested positive for COVID‐19 4 days before his admission.

Upon admission, his vital signs showed a blood pressure of 204/109 mmHg, a heart rate of 104 beats per minute, an axillary body temperature of 39.7°C, and a respiratory rate of 22 breaths per minute. His weight was 87 kg, height was 1.72 m, and body mass index was 29.4 kg/m^2^. Oxygen saturation was 87% on room air. Laboratory investigations revealed a blood urea nitrogen level of 21 mg/dL (normal: 6–20 mg/dL), potassium of 4.0 mmol/L (normal: 3.6–5.2 mmol/L), and sodium of 139 mEq/L (normal: 135–145 mEq/L). Fasting blood glucose was 104 mg/dL (normal: 100–126 mg/dL). The lipid profile showed high‐density lipoprotein of 47 mg/dL (normal: 40–60 mg/dL), low‐density lipoprotein of 119 mg/dL (normal: 100–129 mg/dL), and triglycerides of 133 mg/dL (normal: < 150 mg/dL). Hemoglobin was 14.1 g/dL (normal: 13.8–17.2 g/dL), hematocrit was 42.3% (normal: 41–50%), and serum creatinine was 1.2 mg/dL (normal: 0.7–1.3 mg/dL).

Urinalysis revealed urinary crystals. The white blood cell count was 12,190 cells/mm^3^ (normal: 4500–11,000 cells/mm^3^), with neutrophils of 65% (normal: 55%–70%) and lymphocytes of 37% (normal: 20%–40%). Troponin levels were within the normal range. His electrocardiogram (ECG) revealed left axis deviation, sinus tachycardia, and ST‐segment elevation. A brain computed tomography (CT) scan showed no hypodense lesions suggestive of cerebral infarction and no hyperdense areas indicating intracranial hemorrhage. Brain Magnetic Resonance Imaging (MRI) revealed no evidence of vasogenic edema or other acute intracranial abnormalities. Chest x‐ray demonstrated mediastinal widening with diffuse alveolar infiltrates. Echocardiography revealed a visible intimal flap and thickened ventricular walls. Abdominal CT showed no evidence of renal hypoenhancement suggestive of renal ischemia. Reverse transcription polymerase chain reaction testing of a nasopharyngeal swab was positive for SARS‐CoV‐2. The patient was subsequently transferred to the intensive care unit. Based on clinical and diagnostic findings, he was diagnosed with hypertensive emergency in the context of COVID‐19.

## 4. Therapeutic Regimen

He was receiving oxygen via a nasal cannula at 4 L/min in the intensive care unit. His oxygen saturation increased from 87% to 95% within 45 min. This improvement likely reduced sympathetic overactivity, which can elevate blood pressure and heart rate. Oxygen therapy did not require adjustments in antihypertensive medications but facilitated safer and more effective blood pressure management. He also received fluid resuscitation with intravenous normal saline at 20 mL/kg over the first 4 h. Hydralazine (5 mg) was administered intravenously as the initial antihypertensive agent. It is a rapidly acting vasodilator commonly used to manage acute severe hypertension. In this clinical setting, continuous intravenous infusion agents were not immediately available. IV hydralazine is readily accessible and frequently used for rapid reduction of markedly elevated blood pressure. The patient was re‐evaluated 20 min after IV hydralazine to assess the blood pressure response and determine the need for additional intervention.

Continuous cardiac monitoring was initiated at the start of IV hydralazine and maintained until hemodynamic stabilization. This allowed timely detection of reflex tachycardia, a compensatory response to rapid vasodilation that can increase cardiovascular stress, particularly in COVID‐19. After two doses of IV hydralazine, the patient developed reflex tachycardia. To control heart rate, the patient′s treatment was switched from IV hydralazine to oral atenolol (50 mg once daily), a *β*1‐blocker that reduces cardiac output and prevents hydralazine‐induced reflex tachycardia. The patient also began taking sustained‐release nifedipine (20 mg orally twice daily). By targeting vascular resistance (via nifedipine) and cardiac output (via atenolol), the combination therapy effectively reduce blood pressure.

Alternative IV antihypertensive agents, such as labetalol, nicardipine, and sodium nitroprusside, are recommended for hypertensive emergencies. However, limited availability and the need for close monitoring may restrict their use. IV hydralazine is typically administered at doses of 5–10 mg. Blood pressure is reassessed after 20–30 min, and the dose is titrated according to the patient′s response. In addition, the patient received subcutaneous enoxaparin (80 mg once daily) to prevent thrombotic complications in hospitalized COVID‐19 patients. He was given azithromycin (500 mg once daily for 5 days) to prevent secondary bacterial infections. Acetaminophen (500 mg) was administered as needed to manage fever.

## 5. Follow‐Up and Clinical Course

The patient remained hospitalized for 17 days. During this time, his blood pressure gradually decreased from 204/109 to 142/87 mmHg, and his heart rate stabilized. Target‐organ function also improved. This included normalization of ECG changes, stable renal function, and preserved neurological status. Supportive COVID‐19 therapies were completed. He was discharged after two consecutive negative RT‐PCR tests. At discharge, he was instructed to continue oral antihypertensive medications, including nifedipine and atenolol. He was also advised to maintain monthly follow‐up at the cardiac clinic for blood pressure monitoring and further cardiovascular evaluation (Table 1). At 1‐ and 3‐month follow‐up, his blood pressure remained within the range (130–140/80–90 mmHg) with no cardiovascular complications.

**Table 1:** Timeline of hemodynamic and clinical responses in a 67‐year‐old with hypertensive emergency and COVID‐19.

Time/event	Intervention	Dose/route	BP (mmHg)	HR (bpm)	Oxygen saturation (%)	Clinical response
Admission (ER)	—	—	204/109	104	87 (room air)	Shortness of breath, dizziness, nausea, blurred vision, weakness, lower limb edema. Oxygen therapy started via nasal cannula at 4 L/min; oxygenation improved but hemodynamics unchanged.
+45 min	Oxygen therapy	Nasal cannula 4 L/min	204/109	104	95	Oxygen saturation normalized to normal range; BP and HR remained elevated.
ICU admission	Fluid resuscitation	0.9% normal saline	202/108	102	95	Slight BP reduction and HR decrease after fluids; oxygenation stable. COVID‐19 infection confirmed; ICU monitoring initiated.
	IV hydralazine (1st dose)	5 mg IV	204/109 → 180/100 after 20 min	104 → 110	95	Rapid‐acting vasodilator administered for acute severe hypertension; moderate BP reduction achieved. Reflex tachycardia observed.
20 min post 1st dose	IV hydralazine (2nd dose)	5 mg IV	180/100 → 170/95 after 20 min	110 → 115	95	BP decreased moderately; reflex tachycardia persisted, indicating need for HR control.
After hydralazine	Oral atenolol	50 mg once daily	170/95 → 150/90 after 2 h	115 → 90	97	Atenolol initiated to control HR; successfully resolved hydralazine‐induced reflex tachycardia while maintaining BP reduction.
After atenolol	Oral nifedipine SR	20 mg twice daily	150/90 → 142/87 after 6 h	86	95	Nifedipine added to reduce vascular resistance; combination with atenolol prevented reflex tachycardia. BP lowered effectively.
Hospital stays	Enoxaparin	80 mg SC once daily	141/88	82	96	Prophylaxis against thrombotic complications in COVID‐19; BP and HR stable.
	Azithromycin	500 mg once daily for 5 days	141/88	82	96	Prevent secondary bacterial infections; BP, HR, and oxygen saturation remained stable.
	Acetaminophen	500 mg as needed	141/88	82	96	Fever control (39.7°C on admission); hemodynamics stable.
Day 17	Discharge	—	142/87	79	98	Two consecutive negative COVID‐19 swabs. Patient discharged with instructions to continue nifedipine and maintain monthly follow‐up.
Postdischarge (ambulatory follow‐up)	Long‐term BP management	Nifedipine + enalapril + hydrochlorothiazide	Monitored monthly; expected 130/80–140/90	Monitored; expected 70–78	—	The patient was followed up to monitor BP, ensure adherence, and prevent hypertensive emergencies. He was counseled on lifestyle measures, including diet, weight management, and physical activity. No complications were reported at discharge.

## 6. Clinical Outcome

The patient′s recovery of organ function closely correlated with the controlled reduction of blood pressure to the target range (130–140/80–90 mmHg). Signs of acute stress on the heart, kidneys, brain, and lungs improved steadily (Table 2). This highlights that early recognition and timely management of severe hypertension can prevent further organ injury. The case emphasizes the importance of careful hemodynamic monitoring and individualized treatment, particularly in patients with hypertensive crises during COVID‐19.

**Table 2:** Blood pressure management and resolution of organ dysfunction.

Intervention	BP (mmHg)	HR (bpm)	Organ function/resolution
IV Hydralazine 5 mg two times	204/109 → 170/95	104 → 110 (reflex tachycardia)	Initial reduction of BP; and reflex tachycardia noted.
Oral atenolol 50 mg	170/95 → 150/90	110 → 90	HR controlled, mitigating cardiac stress from reflex tachycardia. ECG changes began to normalize over next 24–48 h.
Oral nifedipine 20 mg SR × 2	150/90 → 142/87	86	Further BP reduction; vascular resistance decreased, and promoting improved perfusion to organs.
Oxygen therapy 4 L/min	—	—	SpO_2_ improved to 95% within 45 min; and hypoxemia resolved.
Hospital course	130–140/80–90	80–90	Echocardiogram at follow‐up showed stabilization of ventricular wall thickness; renal function remained within normal limits (creatinine 1.0 mg/dL); no neurological deficits developed; and respiratory function normalized.

## 7. Discussion

Since April 2024, the COVID‐19 pandemic has affected over 704 million people worldwide. Total reported deaths reached approximately 7,010,681 [10]. The complex effects of COVID‐19 on the cardiovascular and renal systems can markedly worsen hypertension in individuals with preexisting high blood pressure. [11] The virus has affected millions of people worldwide, [12] and certain risk factors increase the likelihood of severe illness. These include advanced age, hypertension, cancer, previous cerebrovascular disease, cardiovascular disease, chronic kidney disease, diabetes mellitus, and obesity. Hypertension, in particular, is one of the most common comorbidities among patients with COVID‐19 [5].

Hospitalized patients with SARS‐CoV‐2 infection who have hypertension are at higher risk of severe clinical outcomes. Specifically, hypertension is associated with increased ICU admissions, worsening heart failure, and higher mortality [13]. Target‐organ damage may involve the heart, brain, eyes, and kidneys. Examples include cardiac ischemia, advanced retinopathy, hypertensive encephalopathy, acute stroke (ischemic or hemorrhagic), acute cardiogenic pulmonary edema, acute aortic disease, eclampsia, and severe pre‐eclampsia/HELLP syndrome (hemolysis and elevated liver enzymes) [14]. In this patient, target‐organ damage included myocardial infarction, as evidenced by ST‐segment elevation, and acute coronary syndrome due to increased cardiac output and oxygen demand. Left ventricular failure, manifested as acute pulmonary edema, and left ventricular hypertrophy, indicated by left axis deviation, were also present.

**Figure 1:**
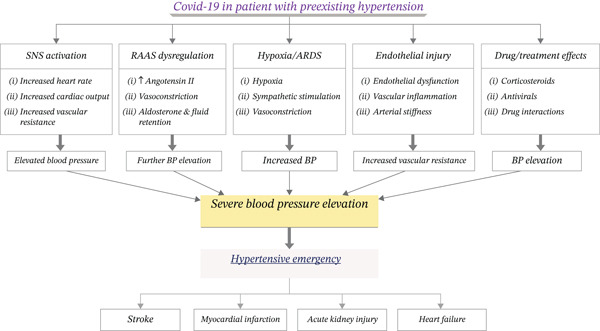
Mechanism of COVID‐19 and preexisting hypertension to driven hypertensive emergencies.

COVID‐19 can trigger hypertensive emergencies through multiple mechanisms (Figure 1). Increased SNS activity can further elevate already high blood pressure. Preexisting cardiovascular changes, such as arterial stiffness and left ventricular hypertrophy, increase vulnerability to SNS overactivity [15]. Dysregulation of the RAAS can amplify angiotensin II‐mediated vasoconstriction, raise blood pressure, and stress organs such as the heart and kidneys [16]. Severe hypoxia, vasoconstriction, and elevated cardiac output associated with COVID‐19 can further increase blood pressure, especially if hypertension is not well controlled [17]. Long‐standing hypertension already stresses the heart and kidneys. COVID‐19–related inflammation, hypoxia, and RAAS dysregulation can lead to acute declines in cardiac and renal function. Acute end‐organ damage, including stroke, myocardial infarction, or renal failure, may occur [18]. Treatments for COVID‐19, such as corticosteroids or antivirals, can also raise blood pressure, potentially precipitating a hypertensive emergency if not carefully monitored [19].

Chronic hypertension causes arterial stiffness and endothelial dysfunction, which may worsen with COVID‐19–induced endothelial injury [20]. Compensatory responses to ARDS, including vasoconstriction and elevated heart rate, can further increase blood pressure and risk of hypertensive crises [21]. In this patient, the hypertensive emergency likely involved RAAS dysregulation and inflammation‐mediated endothelial dysfunction. Although direct RAAS or cytokine assays were not performed, several indirect markers support these mechanisms. The patient exhibited rapid blood pressure elevations, leukocytosis (12,190 cells/mm^3^), neutrophilia (65%), and fever (39.7°C), indicating systemic inflammation and endothelial stress. Cardiac strain on ECG and echocardiography, along with mild renal abnormalities, further suggest end‐organ stress consistent with RAAS activation and inflammation.

Prognosis in hypertensive emergencies among patients with COVID‐19 and preexisting hypertension depends on the extent of organ damage, promptness and efficacy of medical care, and overall health status. Hypertensive emergencies triggered by COVID‐19 often have worse outcomes due to systemic inflammation, higher risk of multiorgan failure, and challenges in controlling both conditions simultaneously [22]. In most hypertensive emergencies, mean arterial pressure should be reduced gradually. The initial reduction is 10–20% during the first hour, followed by an additional 5%–15% over the next 23 h. During the first hour, systolic and diastolic pressures should be lowered to approximately 180 and 120 mmHg, respectively. Blood pressure should then be further reduced to about 160/110 mmHg over the next 23 h [23]. The site of organ damage largely determines the choice of antihypertensive medications, target blood pressure, and timeframe for reduction [24]. Interactions among the cardiovascular, renal, and respiratory systems require careful consideration when managing hypertensive emergencies in patients with COVID‐19 and preexisting hypertension. Management of COVID‐19 may include antiviral therapy, oxygen supplementation, corticosteroids (e.g., dexamethasone in severe cases), and anticoagulation when indicated [25].

Intravenous antihypertensives for hypertensive emergencies include multiple classes. Nicardipine starts at 5 mg/h and is increased by 2.5 mg every 5 min to a maximum of 15 mg/h. Clevidipine begins at 1–2 mg/h, doubling every 90 s until the target, then titrated every 5–10 min up to 32 mg/h for a maximum of 72 h. Sodium nitroprusside is initiated at 0.3–0.5 *μ*g/kg/min and increased by 0.5–10 *μ*g/kg/min, with thiosulfate if infusion exceeds 30 min [25, 26]. Nitroglycerin starts at 5 *μ*g/min and is increased by 5 *μ*g/min every 3–5 min to a maximum of 20 *μ*g/min. Hydralazine is given as a 10 mg IV slow infusion every 4–6 h (maximum 20 mg). Esmolol has a 500–1000 *μ*g/kg/min loading dose over 1 min, followed by a 50 *μ*g/kg/min infusion. Labetalol is 0.3–1.0 mg/kg IV every 10 min (maximum 20 mg) or 0.4–1.0 mg/kg/h infusion up to 3 mg/kg/h, with a cumulative maximum of 300 mg [25]. Phentolamine is given as 5 mg IV every 10 min as needed. Enalaprilat starts at 1.25 mg IV over 5 min, up to 5 mg every 6 h. Fenoldopam begins at 0.1–0.3 *μ*g/kg/min, increased by 0.05–0.1 *μ*g/kg/min every 15 min to a maximum of 1.6 *μ*g/kg/min. [25, 26].

In this patient, concurrent COVID‐19 therapies, including azithromycin, prophylactic enoxaparin, and supplemental oxygen, were administered according to standard care. Corticosteroids, which can elevate blood pressure through fluid retention, were not administered because the patient did not exhibit severe systemic inflammation. The patient′s antihypertensive regimen began with intravenous hydralazine (5 mg), administered immediately. Blood pressure and heart rate were closely monitored, and the patient was re‐evaluated 20 min after administration. After two doses, he developed reflex tachycardia, a compensatory response to rapid vasodilation. To control heart rate, IV hydralazine was switched to oral atenolol (50 mg once daily). Sustained‐release nifedipine (20 mg orally twice daily) was also initiated. Medications were titrated according to blood pressure and heart rate. This effectively controlled the hypertensive emergency and attenuated reflex tachycardia. Potential drug–drug interactions were considered.

Hydralazine and atenolol posed no significant interactions with the COVID‐19 therapies. Nifedipine, metabolized via CYP3A4, could theoretically interact with azithromycin, a weak CYP3A4 inhibitor; however, no clinically relevant effects were observed. Recent evidence suggests that the incidence of new‐onset hypertension has increased during and after the COVID‐19 pandemic [27]. This is likely related to endothelial dysfunction, systemic inflammation, and dysregulation of the RAAS. These findings highlight the importance of careful monitoring and management of hypertensive emergencies in COVID‐19 patients.

## 8. Conclusion

This case highlights the importance of early recognition and timely management of hypertensive emergencies in patients with COVID‐19. Rapid blood pressure control using intravenous hydralazine, followed by oral atenolol and nifedipine, effectively reduced both blood pressure and reflex tachycardia. This approach prevented further target‐organ injury. Continuous monitoring, appropriate supportive care, and attention to potential drug interactions are essential for optimizing outcomes. Maintaining long‐term blood pressure control and regular follow‐up is critical to prevent recurrent hypertensive crises, particularly for patients with preexisting hypertension who also have COVID‐19.

## Funding

No funding was received for this manuscript.

## Disclosure

A preprint version of this manuscript was previously published by (Bereda G. [2025]) [28] on MDPI prepr http://ints.org/ with Doi: 10.20944/preprints202503.2236.v1.

## Ethics Statement

Our institution does not require ethical approval for reporting individual cases or case series.

## Consent

Written informed consent was obtained from the patient for publication of this case report.

## Conflicts of Interest

The author declares no conflicts of interest.

## Data Availability

Data sharing is not applicable to this article as no datasets were generated or analyzed during the current study.
